# Effects of wearable resistance training on linear sprinting and jumping abilities in healthy populations: a systematic review and three-level meta-analysis

**DOI:** 10.3389/fphys.2025.1590866

**Published:** 2025-06-18

**Authors:** Chen Wei, Zihan Zhou, Fuhai Wang, Xiaoquan Zhang

**Affiliations:** ^1^ Shenyang Normal University, College of Sports Science, Shenyang, China; ^2^ Beijing Normal University, College of P.E and Sports, Beijing, China

**Keywords:** wearable resistance training, sprint, jump, biomechanics, meta-analysis

## Abstract

**Objective:**

This study aimed to systematically evaluate and analyze the effects of weighted resistance training (WRT) on linear sprinting and jump abilities in healthy populations through a three-level meta-analysis.

**Methods:**

We systematically searched five databases, including PubMed, Web of Science, The Cochrane Library, SPORTDiscus, and Embase, for randomized controlled trials (RCTs) investigating the effects of WRT on linear sprinting and jumping abilities, with the search conducted from database inception until 1 May 2025. The quality of the literature was assessed using the Cochrane ROB2 tool and the Physiotherapy Evidence Database (PEDro) scale, whereas the quality of evidence was evaluated using GRADE. A three-level random effects model was implemented in R for the meta-analysis, along with an assessment of publication bias. Hedges’ g and its 95% confidence intervals (CIs) were calculated for evaluation. Publication bias was examined using funnel plots and multilevel Egger’s regression tests.

**Results:**

Ten studies, comprising 256 participants, were included. The meta-analysis results indicated that WRT significantly improved linear sprinting ability (95% CI: −0.558 to −0.027, p < 0.05), while no significant effect was observed on jumping ability (95% CI: −0.067 to −0.545, p = 0.118). Subgroup analyses revealed that WRT positively effected 10-m linear sprinting performance (g = −0.393, 95% CI -0.784 to −0.002, p = 0.049). Specifically, trunk WRT (g = −0.554, 95% CI -1.013 to −0.096, p = 0.020) and weights ≤10% of body mass (BM) (g = −0.495, 95% CI -0.884 to −0.107, p = 0.014) significantly improved sprinting performance. The GRADE assessment indicated that the quality of evidence regarding the effects of WRT on linear sprinting and jumping abilities was low.

**Conclusion:**

These findings suggest that WRT with trunk load and weights ≤10% of BM can enhance start acceleration (0–10 m) during linear sprinting. However, WRT did not significantly improve jumping ability.

## 1 Introduction

In many sports, rapid short-distance sprinting ability and exceptional jumping ability are crucial factors for success in competitions (Macadam et al., 2019; Baena-raya et al., 2022). To achieve this, athletes often engage in strength training to enhance their linear sprinting and jumping abilities (Cronin et al., 2014). Previous studies have demonstrated a positive correlation between strength and sprinting ability (r = 0.49) as well as jumping ability (r = 0.54) (Swinton et al., 2014; Suchomel et al., 2016). Traditional resistance training may be an appropriate method for enhancing maximal strength in athletes; however, it may not be the optimal choice for strength training that also requires speed enhancement (Simperingham et al., 2022). Additionally, the training environment for traditional strength training often differs from the competitive environment encountered by athletes during competitions (Bustos et al., 2020). Therefore, ensuring that technical movement patterns are specialized while providing appropriate load stimuli to the target muscle groups is crucial for bridging training and competition. Moreover, developing a training program that provides sufficient training volume within a limited timeframe and effectively translating the strength gains from resistance training into athletic performance has become a new challenge (Hurst et al., 2022).

Research has shown that resistance training is one of the most effective methods for developing strength and coordination, enhances central nervous system excitability, induces high-frequency neural impulses to improve muscle fiber recruitment, and has a significant effect on increasing muscle strength and maximal strength (Swinton et al., 2024). Wearable resistance training (WRT) is a training method that applies a certain percentage of body mass (BM) as resistance to specific body parts (such as the trunk, arms, and lower limbs) without affecting movement techniques, thereby integrating resistance training with actual competitive environments (Chua et al., 2021). WRT enhances muscle recruitment and force output through load overload, specifically improving intermuscular coordination during full-range movements, including promoting motor unit recruitment and discharge frequency (Hammett and Hey, 2003). This approach provides specific physiological adaptations for athletes, thereby improving their performance (Macadam et al., 2017a; Macadam et al., 2021). WRT has been widely implemented in the field of competitive sports training as an effective specialized method of resistance training. Acute interventions aim to observe changes in dynamic parameters due to WRT, where acute WRT may affect parameters such as sprinting speed, stride length, and step width, while reducing jumping height and landing forces. On the other hand, long-term interventions assess improvements in athletic performance resulting from WRT. Negra et al. (Negra et al., 2020) indicated that compared to bodyweight jumping training, 8% BM trunk WRT did not show significant improvements in linear sprinting performance, but it significantly enhanced standing long jump (SLJ) results. Other studies have shown that low-weight calf-loaded warm-up training significantly improves linear sprinting and horizontal jumping abilities (Bustos et al., 2020).

A review of previous studies revealed that numerous investigations have reported the effects of long-term WRT on lower limb performance (Bertochi et al., 2024). However, there is currently no clear consensus on the intervention effects, with many conflicting conclusions (Macadam et al., 2022; Gleadhill et al., 2021). This may be related to various factors, such as the weight, form, and targeted areas of WRT, as well as the level of trainees. When investigating the effects of WRT on linear sprinting and jumping abilities, it is common for a single study to report multiple effect sizes such as different distances for linear sprints and various forms of jump performance. In traditional meta-analyses, including multiple effect sizes from the same study violates the principle of effect size independence; however, extracting only the largest effect size from the literature may lead to overly optimistic results (Xu et al., 2024). Therefore, the main objective of this study was to explore the effects of WRT on linear sprinting and jumping abilities through a three-level meta-analysis, aiming to maximize the utilization of original data and optimize statistical efficiency, as well as to identify factors that may influence the intervention effects of WRT on linear sprinting and jumping abilities in healthy populations. This study aimed to provide an objective and scientific summary and recommendations for future WRT applications.

## 2 Methods

This meta-analytical review was conducted in accordance with the Preferred Reporting Items for Systematic Reviews and Meta-Analyses statement and was registered in an international database of systematic reviews in health and social care (registration number: CRD42024619143; https://www.crd.york.ac.uk/PROSPERO/view/CRD42024619143).

### 2.1 Search strategy

To conduct this analysis, five databases including Embase, WOS, PubMed, Cochrane Library, and SPORTDiscus were searched, to collect relevant randomized controlled trials (RCTs), with the search period extending from the inception of the databases to 1 May 2025. Additionally, the references of the included studies were manually searched to ensure that no important literature was overlooked. The literature search strategy is shown in Table 1.

**TABLE 1:** The literature search strategy.

Database	Search strategy
PubMed	#1 (sprint kinematics [Title/Abstract] OR sprint performance [Title/Abstract] OR jump performance [Title/Abstract] OR jump [Title/Abstract] OR jumping ability [Title/Abstract] OR sprinting ability [Title/Abstract] OR linear sprint [Title/Abstract] OR countermovement jump [Title/Abstract] OR squat jump [Title/Abstract] OR CMJ [Title/Abstract] OR SJ [Title/Abstract]) #2 (wearable resistance [Title/Abstract] OR wearable resistance training [Title/Abstract] OR WRT [Title/Abstract] OR extra load [Title/Abstract] OR weighted vest [Title/Abstract]) #3 #1 AND #2
Web of Science	#1:TS=(sprint kinematics OR sprint performance OR jump performance OR jump OR jumping ability OR sprinting ability or linear sprint or countermovement jump or squat jump or CMJ or SJ) #2:TS=(wearable resistance OR wearable resistance training OR WRT OR extra load OR weighted vest) #3:#1 AND #2
Embase	#1′sprint kinematics ':ab,ti OR ' sprint performance ':ab,ti OR ' jump performance ':ab,ti OR ' jump ':ab,ti OR ' jumping ability ':ab,ti OR ' linear sprint ':ab,ti OR ' countermovement jump ':ab,ti OR ' squat jump ':ab,ti OR ' CMJ ':ab,ti OR ' SJ ':ab,ti #2′wearable resistance ':ab,ti OR ' wearable resistance training ':ab,ti OR ' WRT ':ab,ti OR ' extra load ':ab,ti OR ' weighted vest ':ab,ti #3:#1 AND #2
SPORTDiscus	#1:AB=(sprint kinematics OR sprint performance OR jump performance OR jump OR jumping ability OR sprinting ability or linear sprint or countermovement jump or squat jump or CMJ or SJ) #2:AB =(wearable resistance OR wearable resistance training OR WRT OR extra load OR weighted vest) #3:#1 AND #2
Cochrane	#1: (sprint kinematics):ti,ab,kw or (sprint performance):ti,ab,kw or (jump performance):ti,ab,kw or (jump):ti,ab,kw or (jumping ability):ti,ab,kw or (sprinting ability):ti,ab,kw or (linear sprint):ti,ab,kw or (countermovement jump):ti,ab,kw or (CMJ):ti,ab,kw or (SJ):ti,ab,kw #2: (wearable resistance):ti,ab,kw or (wearable resistance):ti,ab,kw or (WRT):ti,ab,kw or (extra load):ti,ab,kw or (weighted vest):ti,ab,kw #3:#1 AND #2#3:#1 AND #2

### 2.2 Inclusion and exclusion criteria

Inclusion criteria: 1. The study subjects were athletes or populations with sports backgrounds. 2. Wearable resistance training was the intervention in the experimental group. 3. The control group underwent the same training method as the experimental group but without an added load. 4. Outcome measures included either linear sprinting or jumping. 5. The literature must be peer-reviewed, randomized controlled trials (RCTs). 6. The language type was English.

Exclusion criteria: 1. Studies on acute WRT. 2. Inability to access full-text or extract data. 3. Reviews or conference abstracts. 4. Outcome measures that did not include linear sprinting or jumping. 5. Inconsistent intervention types. 6. Population mismatch, such as obese, frail, or sedentary individuals.

### 2.3 Literature screening and data extraction

After retrieving the relevant literature, we imported the documents into the Endnote X20 software for deduplication. Two researchers independently screened the literature titles and abstracts in a double-blind manner and extracted the data according to a pre-designed table. In case of any disagreement, a third researcher participated in the discussion to determine whether to include the study. Extracted information included the first author’s name, publication year, baseline characteristics of the study subjects (age, sex, height, weight, and sport), training methods, and outcome measures.

### 2.4 Quality evaluation

The included studies were assessed by two reviewers using the Cochrane Collaboration risk of bias (RoB) 2.0 tool (Sterne et al., 2019) and Physiotherapy Evidence Database (PEDro) scale (Yin et al., 2025). The ROB2 evaluates biases related to randomization, intervention, missing outcome data, outcome measurement, and selective reporting. The risk of bias in each domain was rated as “low risk,” “some concerns,” or “high risk.”

The PEDro scale consists of 11 items: eligibility criteria, random allocation, concealed allocation, baseline comparability, participant blinding, therapist blinding, assessor blinding, follow-up of >85%, intention-to-treat analysis, between-group statistical analysis, point estimates, and variability measures. Scores ranging from 0 to 10 were assigned based on the fulfillment of each item, with 1 point indicating meeting the criteria and 0 points indicating not meeting the criteria or unclear information. Studies were classified as high-quality (9–10), moderately high-quality (6–8), satisfactory (4–5), or low-quality (<4) based on their total score.

The GRADE system was also used to assess the quality of evidence for the outcome measures (Yin et al., 2025), categorizing quality into four levels: high, moderate, low, and very low. Two researchers independently conducted quality assessments based on the evaluation criteria. If discrepancies arose, a third researcher participated in the discussion until a consensus was reached.

### 2.5 Statistical analysis

Analyses were conducted using the R software. A three-level meta-analysis with a random-effects model was employed to manage the dependency of effect sizes within studies (Wilson et al., 2016; Cui et al., 2024). Standardized mean differences and their variances were calculated based on the post-test means, standard deviations, and sample sizes of the experimental and control groups. The Hedges’ g value and its 95% confidence interval (CI) were used for assessment, where g ≤ 0.20 indicates a small effect, 0.20–0.49 indicates a small to moderate effect, 0.5–0.79 indicates a moderate effect, and g ≥ 0.80 indicates a large effect (Sheline et al., 1996). Statistical significance was defined as P < 0.05. Funnel plots and three-level Egger’s regression tests were used to assess publication bias.

## 3 Results

### 3.1 Literature search results

**FIGURE 1:**
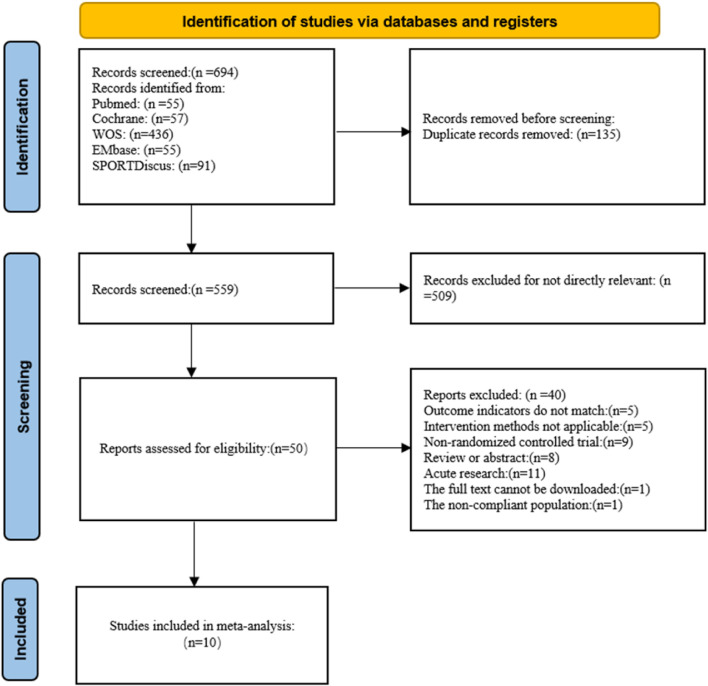
Flowchart of the study selection process.

A total of 694 relevant studies were identified. After excluding 135 duplicate publications, titles and abstracts were reviewed, resulting in the exclusion of 509 unrelated studies. Full-text studies were reviewed, leading to the exclusion of 5 studies with incompatible outcome measures, 5 studies with incompatible intervention methods, 9 non-randomized controlled trials, 9 reviews or abstracts, 11 acute studies, 1 study that could not be downloaded, and 1 study that the participants do not meet the criteria. Ultimately, 10 studies were included (Figure 1) (Bustos et al., 2020; Negra et al., 2020; Barr et al., 2015; Khlifa et al., 2010; Markovic et al., 2013; Marriner et al., 2017; Rey et al., 2017; Ryan et al., 2025; Feser et al., 2021; Rodríguez-Osorio et al., 2019).

### 3.2 Quality evaluation of the literature included

**FIGURE 2:**
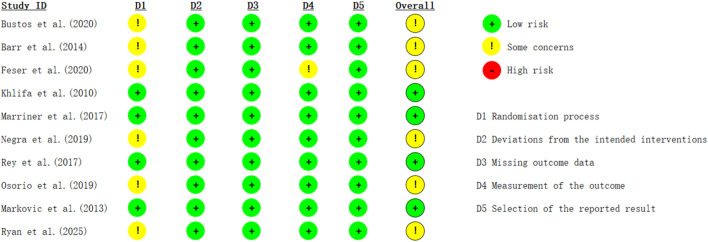
Methodological assessments by the RoB2.0 tool.

All 10 included studies were randomized controlled. Among them, 6tudies provided detailed descriptions of the randomization process and exhibited low risk of bias.10tudies described the planned interventions, also showing low risk of bias.10 studies had no missing outcome data, thus posing no bias.9 studies showed no bias in outcome measurement.10 studies exhibited no bias in the selection of reported outcomes. The final quality rating for the included studies was 6 studies classified as moderate-quality literature and 4 studies classified as high-quality literature (Figure 2). Two independent reviewers conducted literature screening during the inclusion process and obtained a Cohen’s kappa value of 0.82. For both the researchers indicated a high level of agreement. The average PEDro score of all the studies was 6.4, indicating that the methodological quality of the included studies was generally moderate to high (Table 2).

**TABLE 2:** Methodological assessments by the PEDro.

Study	1	2	3	4	5	6	7	8	9	10	11	TS
Bustos et al. (2020)	Y	Y	N	Y	N	N	N	Y	Y	Y	Y	6
Barr et al. (2015)	Y	Y	N	Y	N	N	N	Y	Y	Y	Y	6
Feser et al. (2021)	Y	Y	N	Y	N	N	N	Y	N	Y	Y	5
Khlifa et al. (2010)	Y	Y	Y	Y	N	N	N	Y	Y	Y	Y	7
Marriner et al. (2017)	Y	Y	Y	Y	N	N	N	Y	Y	Y	Y	7
Negra et al. (2020)	Y	Y	N	Y	N	N	N	Y	Y	Y	Y	6
Rey et al. (2017)	Y	Y	Y	Y	N	N	N	Y	Y	Y	Y	7
Rodríguez-Osorio et al. (2019)	Y	Y	Y	N	N	N	N	Y	Y	Y	Y	6
Markovic et al. (2013)	Y	Y	Y	Y	N	N	N	Y	Y	Y	Y	7
Ryan et al. (2025)	Y	Y	Y	Y	N	N	N	Y	Y	Y	Y	7
Mean score												6.4

1 Eligibility criteria, 2 allocation of randomization, 3 concealed allocation, 4 similarity baseline, 5 subject blinding, 6 therapist blinding, 7 assessor blinding, 8 more than 85% retention, 9 intention-to-treat analysis, 10 between-group comparisons, 11 point and variability measures, TS, total score, Y explicitly described and presented in detail, N absent, inadequately described or unclear.

### 3.3 Basic characteristics of included studies

Ten studies were included, all of which were RCTs. The study included 256 participants, with 139 in the experimental group and 117 in the control group. Among the 10 studies, nine involved male participants and one involved female participants. The studies included four targeting football players, two targeting rugby players, one targeting basketball players, one targeting volleyball players, and two focusing on the general exercise population. Regarding the WRT loading sites, seven studies focused on trunk loading, whereas three focused on lower limb loading. The weight range for WRT varied from 200 g to 50% of BM, and the training methods included warm-up training, jump training, change of direction (COD) training, strength and speed training, power cleaning training, and sprint training. Seven studies assessed linear sprinting ability, whereas eight studies evaluated jumping ability. The basic characteristics of the included studies are presented in Table 3.

**TABLE 3:** Basic characteristics of included studies.

Study	N (E.G.,/CG)	Gender (Male/Fmale)	Age (years) (E.G.,/CG)	Height (E.G.,/CG)	BM(kg) (EG/CG)	Population	Load position	Load weight	Duration	Forms of WRT	Assessment
Bustos et al. (2020)	15/16	31/0	17.1 ± 0.76	176 ± 0.61 cm	68.5 ± 5.42	Soccer Athletes	Lower limb	200–600 g	8w	Warm up	10 m,20 m,CMJ,SLJ
Barr et al. (2015)	8/7	31/0	22.4 ± 2.7/22.0 ± 2.1	1.82 ± 0.06/1.86 ± 0.07(m)	95.3 ± 7.1/92.8 ± 11.4	Rugby Athletes	Trunk	12%BM	8d	Strength and Speed Training	10 m,40 m
Feser et al. (2021)	12/10	32/0	22.6 ± 2.94/24.6 ± 2.99	182.6 ± 8.60/178.8 ± 5.69 (cm)	96.5 ± 13.6/92.5 ± 12.9	Rugby Athletes	Lower limb	1%BM	6w	Sprint Training	5 m,10 m,20 m,30 m
Khlifa et al. (2010)	9/9	18/0	23.11 ± 0.32/24.16 ± 0.19	193.18 ± 0.77/192.58 ± 0.86 (cm)	83.13 ± 0.70/82.61 ± 0.79	Basketball Athletes	Trunk	10%∼11%BM	10w	Jump Training	CMJ,SJ
Marriner et al. (2017)	8/8	16/0	23.1 ± 2.3/23.3 ± 3.8	N/A	93.9 ± 11.0/87.2 ± 9.8	Physically active males	Trunk	12%BM	5w	Power clean training	CMJ
Negra et al. (2020)	13/16	29/0	13.0 ± 0.7/13.0 ± 0.5	162.6 ± 8.3/159.6 ± 11.6 (cm)	45.7 ± 8.0/42.4 ± 8.8	Soccer Athletes	Trunk	8%BM	8w	Jump Training	5 m,10 m,20 m,CMJ
Rey et al. (2017)	10/9	19/0	23.6 ± 2.7/23.7 ± 2.1	178.5 ± 4.9/179.8 ± 4.8 (cm)	73.9 ± 6.5/75.1 ± 6.8	Soccer Athletes	Trunk	18.90%BM	6w	Sprint Training	10 m,20 m,CMJ
Rodríguez-Osorio et al. (2019)	EG1:19 EG2:19 CG:16	44/0	EG1:18.8 ± 5.3 EG2:17.7 ± 3.4 CG:17.8 ± 4.2	EG1:174.2 ± 8.1 EG1:173.5 ± 6.1 CG:164.8 ± 4.0 (cm)	EG1:63.9 ± 11.5 EG1:63.2 ± 8.1 CG:64.7 ± 9.2	Soccer Athletes	Trunk	EG1: 12.5% BM EG2: 50%BM	6w	COD Training	10 m,20 m,30,CMJ
Markovic et al. (2013)	11/12	33/0	23.7 ± 1.7	182.4 ± 6.1/182.8 ± 6.0 (cm)	81.0 ± 8.0/82.5 ± 8.7	Physical education students	Trunk	30%BM	8w	Jump Training	CMJ,SJ
Ryan et al. (2025)	15/14	0/29	15.8 ± 0.68/16.14 ± 0.95	170.06 ± 4.10/170.73 ± 7.92 (cm)	66.85 ± 10.92/64.66 ± 10.88	Netball Athletes	Lower limb	1%∼1.5%BM	6w	Warm up	5 m,15 m,SLJ,CMJ

E.G.,, experimental group; CG, control group; N/A = not available; CMJ, countermovement jump; SLJ, standing long jump; SJ, squat jump; 5 m、10 m、20 m、30 m、40 m indicates different sprint distances.

### 3.4 Meta-analysis

#### 3.4.1 The effect of WRT on linear sprinting ability

**FIGURE 3:**
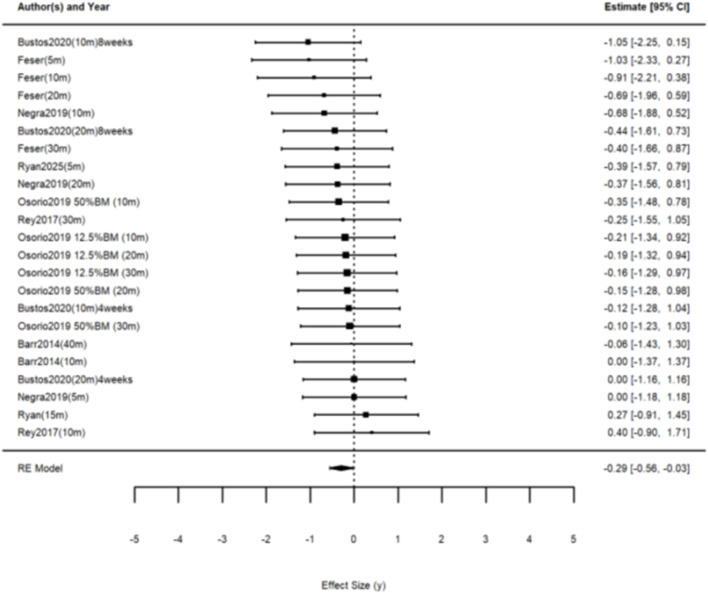
The forest plot of the effect of WRT on linear sprinting ability.

Seven studies were included, yielding twenty-three effect sizes to investigate the effect of WRT on linear sprinting ability. The results of the three-level meta-analysis indicated an effect size of g = −0.292 (95% CI: −0.558 to −0.027, p < 0.05), with Q (df = 22) = 7.655, p = 0.998, indicating nonsignificant heterogeneity (Figure 3). Compared to the control group, WRT significantly improved linear sprinting ability in the healthy population.

#### 3.4.2 The effect of WRT on jumping ability

**FIGURE 4:**
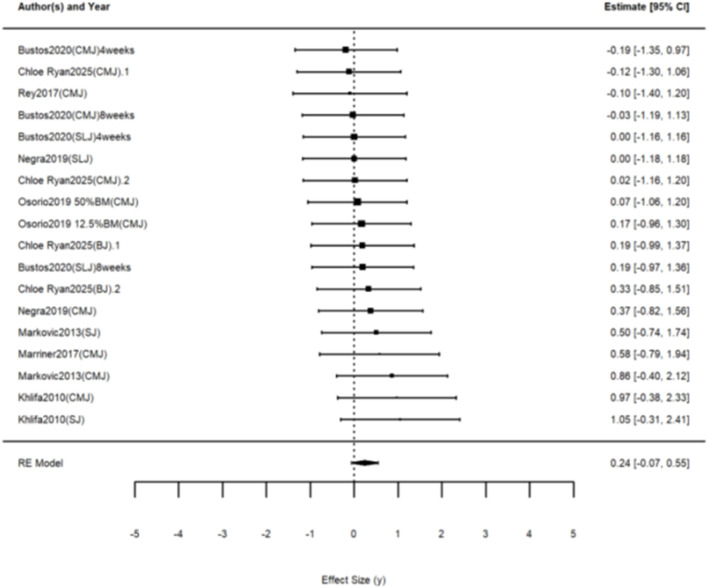
The forest plot of the effect of WRT on jumping ability.

Eight studies were included, yielding 18 effect sizes to investigate the effect of WRT on jumping ability. The results of the three-level meta-analysis indicated an effect size of g = 0.238 (95% CI: −0.067 to 0.545, p = 0.118), with Q (df = 17) = 5.797, p = 0.994, indicating non-significant heterogeneity (Figure 4). Compared to the control group, WRT did not significantly improve jumping ability in the healthy population.

### 3.5 Influence analysis

**FIGURE 5:**
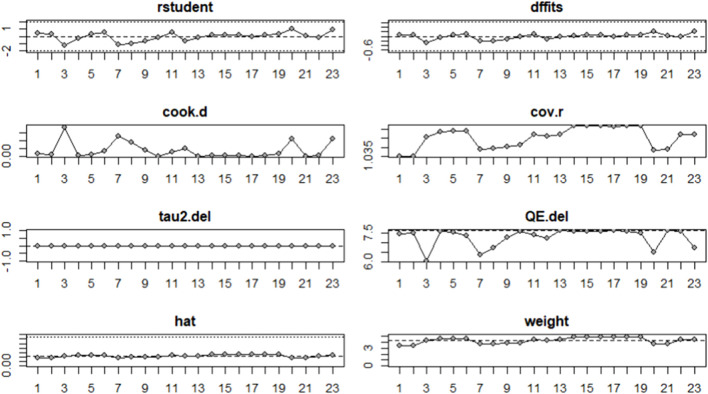
Influence analysis of linear sprinting ability.

**FIGURE 6:**
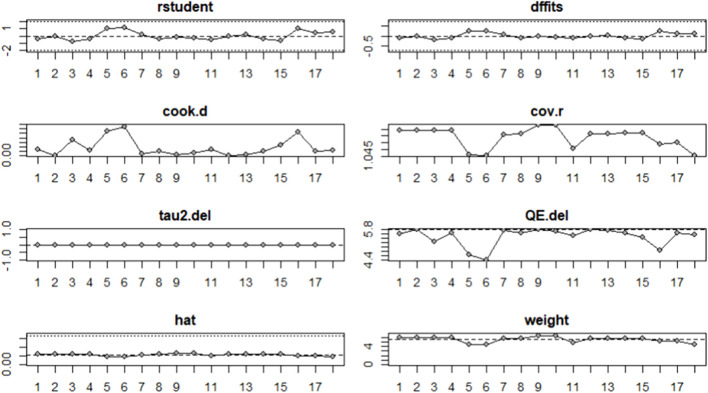
Influence analysis of jumping ability.

To examine whether outliers affected the results of the meta-analysis, influence analyses were conducted for linear sprinting ability (Figure 5) and jumping ability (Figure 6); the results showed no significant outliers.

### 3.6 Subgroup analysis

To investigate whether factors influence the intervention effects of WRT on athletes’ linear sprinting ability, subgroup analyses were conducted based on linear sprinting distances, load position of WRT, and loading weights. The different distances for linear sprints were categorized as 0–10m, 10–20m, 20–30m, 30–40m, and over 40 m; the attachment sites were classified into trunk and lower limbs; and the loading weights were divided into >10% of BM and ≤10% of BM. The results of subgroup analyses are shown in Table 4.

**TABLE 4:** Subgroup analyses of linear sprinting ability.

Outcome measure	Subgroup	k	g (95% CI)	P	Test of Moderators
Linear sprint ability	Sprint distance				F (3,19) = 0.196, p = 0.897
	10 m	11	−0.393 (−0.784, −0.002)	0.049	
	20 m	7	−0.212 (−0.686, 0.261)	0.359	
	30 m	4	−0.215 (−0.854, 0.425)	0.566	
	40 m	1	−0.063 (−1.524, 1.397)	0.928	
	Load position				F (1, 21) = 0.133, p = 0.719
	Trunk	8	−0.554 (−1.013, −0.096)	0.020	
	Lower limb	15	−0.159 (−0.486, 0.167)	0.322	
	Load weight				F (1, 21) = 2.240, p = 0.149
	≤10%	11	−0.495 (−0.884, −0.107)	0.014	
	>10%	12	−0.111 (-0.477, 0.254)	0.533	

k = number of effect sizes; CI, confidence intervals.

### 3.7 Publication bias

**FIGURE 7:**
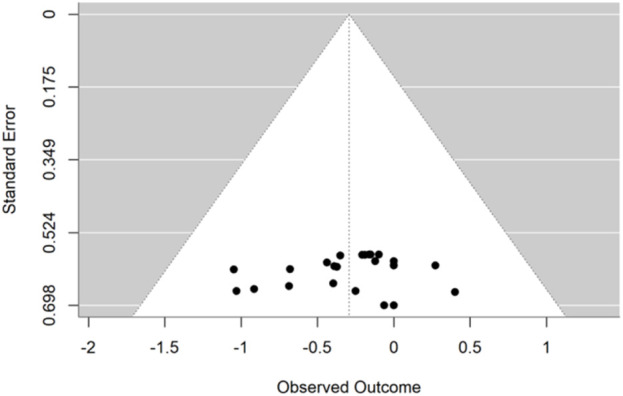
Funnel plot of potential publication bias of linear sprinting ability.

**FIGURE 8:**
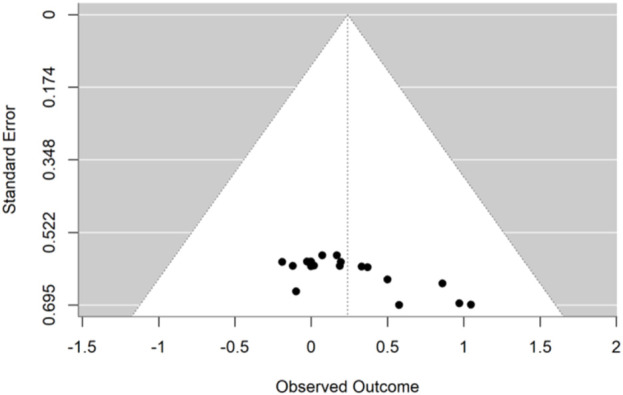
Funnel plot of potential publication bias of jumping ability.

Publication bias was assessed for linear sprinting ability (Figure 7) and jumping ability (Figure 8), and the results of Egger’s test indicated that, for linear sprinting ability (t = −0.406, p = 0.688) and jumping ability (t = 1.742, p = 0.100), there was no significant publication bias in either case.

### 3.8 Evaluation of evidence quality

The quality of evidence for the outcome measures of sprinting and jumping abilities was evaluated, and GRADE analysis indicated that the effect of WRT on both sprinting and jumping abilities was assessed as low-quality evidence (Table 5).

**TABLE 5:** GRADE quality of evidence evaluation.

Outcome measure	Risk of bias	Indirectness	Inconsistency	Imprecision	Publication bias	Quality rating
Linear Sprinting Ability	Serious	Not Serious	Not Serious	Serious	Not Serious	Low
Jumping Ability	Serious	Not Serious	Not Serious	Serious	Not Serious	Low

## 4 Discussion

The results of this study indicate that WRT can significantly improve the linear sprinting ability of healthy populations but does not significantly improve jumping ability. The GRADE analysis rated the quality of evidence regarding the effect of WRT on both linear sprinting and jumping abilities as low. This is attributed to the fact that many of the included studies did not implement allocation concealment, and that the unique nature of WRT makes it difficult to meet the requirements for blinding. Consequently, there may have been a risk of bias in the included studies. Additionally, the sample size of the included studies was relatively small, with a total of 256 participants, which could have contributed to the imprecision in the results. A traditional meta-analysis (Fernández-Galván et al., 2022) demonstrated that trunk-loaded WRT could improve athletes’ linear sprinting performance, but the effect was not significantly superior to that of unresisted sprint training, which is inconsistent with our findings. This discrepancy may be due to the inclusion of multiple effect sizes in the same study. Current research on the effect of WRT on jumping ability has been consistently positive. For instance, Markovic et al. (Markovic et al., 2013) found that the WRT group using 30% BM load for jump training over 8 weeks showed superior training effects in CMJ and SJ compared to unresisted training. Macadam et al.'s systematic review (Macadam et al., 2017a) also indicated that different loading weights (7%–30% BM) improved jumping ability. However, our study, employing a three-level meta-analysis approach, did not find a significant effect of WRT on jumping ability, possibly because of the wide range of loading weights included (200 g - 50% BM). Future research should explore the specific effect of WRT on jumping ability at certain weights.

This study found that concerning linear sprinting ability, WRT had a more pronounced improvement effect on 10 m sprints. Regarding the loading weight, WRT showed a significant improvement when the load was ≤10% BM. In terms of the loading position, trunk WRT demonstrated a more significant improvement in linear sprinting ability. As a form of resistance training, applying loads to different body parts yields varying effects (Carlos-Vivas et al., 2019), which may lead to significant differences in athletic performance. Trunk loading can distribute the load more evenly across the wearer’s center of mass (Macadam et al., 2017b), thereby reducing the interference of the load on the upper and lower limb muscle groups and related kinematic parameters and facilitating more specialized neuromuscular adaptations (Su et al., 2023). However, increasing the load is not always beneficial; some studies indicate that exceeding a certain threshold of loading weight leads to a linear decrease in the running speed (Macadam et al., 2017a). The start phase holds significant importance in sprinting, with elite athletes able to accelerate to one-third of their maximum speed using the start (Sado et al., 2023; Bezodis et al., 2019). The acceleration phase primarily occurred within the first 10 m (Marques and Izquierdo, 2014). Because this phase has a longer ground contact time, the lower limbs need sufficient strength to generate greater propulsion to overcome the inertia caused by the load to increase the running speed (Morin et al., 2011; Rabita et al., 2015; Comfort et al., 2012; Habibi et al., 2010). Trunk WRT primarily optimizes the lengthening and shortening cycles of the lower limb muscles by increasing the vertical load, enhancing the efficiency of the lower limbs in utilizing vertical ground reaction forces, and improving the coordination and stability control of the entire body. This type of training enhances the elastic reserve and power output of the lower limb muscles during sprinting, thereby promoting acceleration ability (Morin et al., 2015; Nuell et al., 2021). Conversely, adding weight to the lower limbs may restrict the range and speed of hip, knee, and ankle joint flexion and extension to some extent, thereby significantly affecting the sprinting technique. This weighted training may limit the functional execution of the hip joint, causing the knee or ankle joints to bear greater local loads and enhancing compensatory movement functions (Chaabene et al., 2018; Dong et al., 2024). Therefore, lower-limb WRT may be more suitable for high-level athletes, focusing on technical details.

WRT is commonly used to enhance athletic performance and can improve sports performance or reinforce specific movement patterns when combined with specialized movement training. However, the reasonable use of WRT in training should depend on the characteristics of the sport and individual needs of the athlete. During WRT, it is essential to arrange the training content appropriately to avoid excessive fatigue, which could lead to a decline in training effectiveness. WRT can be set up with attachments on the trunk or limbs, and different loading positions have varying kinematic and dynamic effects on linear sprinting and jumping performance. More research is needed to conduct horizontal and vertical comparisons of different loading positions.

### 4.1 Strengths and limitations

This study is the first to explore the effect of WRT on linear sprinting and jumping abilities in healthy populations using a three-level meta-analysis method. Although the results indicate that WRT only significantly improves linear sprinting, we have specified that the WRT protocol should focus on trunk loading with a load of ≤10% BM, and that improvements in linear sprinting are notably more significant in the first 10 m. This finding can provide a reference for athletes, coaches, and sports enthusiasts in their training plans. However, this study has some limitations. The loading weight of WRT is related to the weight of the trainees, and factors such as the height and weight of the trainees may significantly influence the effectiveness of WRT on linear sprinting and jumping abilities. Owing to the limited number of included studies, these factors were not discussed in depth. The levels of subjects in the included studies varied, and different athlete disciplines may prioritize the development of linear sprinting and jumping abilities differently, suggesting that the WRT protocol may vary across different levels or sports. Due to the fact that this study only included specific athlete populations and training methods, the external validity of the results may be limited. However, these findings may not be directly applicable to different sports or training backgrounds.

## 5 Conclusion

Based on the results of this study, we can conclude that WRT can enhance the linear sprinting ability of healthy individuals. However, specific training methods should be adjusted according to their own unique abilities. Trunk-loaded WRT is recommended to improve linear sprinting ability, particularly during the start and acceleration phases (0–10 m), trunk-loaded WRT is recommended, with loads ≤10% BM. It is important to note that this study did not demonstrate significant improvement effect of WRT on jumping ability in healthy populations. The findings of this study can provide guidance for athletes, coaches, and sports enthusiasts in implementing resistance training.
